# The Potential Malignancy of a Solitary Fibrous Tumour of the Lung

**DOI:** 10.1155/2015/209490

**Published:** 2015-12-17

**Authors:** Rajeev Shukla, Davide Patrini, Elaine Borg, David Lawrence, Martin Hayward, Nikolaos Panagiotopoulos

**Affiliations:** ^1^Department of Cardiothoracic Surgery, The Heart Hospital, University College London Hospitals (UCLH), London, UK; ^2^Department of Pathology, University College London Hospitals (UCLH), London, UK

## Abstract

Solitary fibrous tumours (SFTs) are rare neoplasms that in the majority of cases are benign. We present the case of a 52-year-old male, with a 23-year history of a slow growing pleural mass, presenting to our department with worsening dyspnoea and localised chest discomfort. The purpose of this case report is to highlight the potential malignancy of a solitary fibrous tumour of the lung along with the key features in diagnosis and management.

## 1. Introduction

Solitary fibrous tumours (SFTs) are rare neoplasms with a reported incidence of 2.8 cases per 100,000 persons per year [1]. SFTs originate from mesenchymal cells and represent less than 5% of all pleural tumours [1–5]. 80% of SFTs arise from the visceral pleura and may present at any age, although being more frequently encountered between the fifth and eighth decades of life [4–8]. SFTs generally have an indolent course and thus are often an incidental finding on chest images [7]. Although the vast majority of SFTs are benign, they do have malignant potential and therefore should be excised [1, 2, 4]. Besides particular histopathological features and immunohistochemical labeling, that is, CD34, CD99, and bcl-2, several studies have shown that SFTs exhibit a specific molecular hallmark demonstrating the gene fusion NAB2-STAT6. This leads to activation of EGR1 and transcriptional decoupling of target genes dependent on EGR-1 and therefore initiating the tumourgenic process [9, 10]. We present the case of a 52-year-old male, with a 23-year history of a slow growing pleural mass, presenting to our department with worsening dyspnoea and localised chest discomfort.

## 2. Case Report

**Figure 1:**
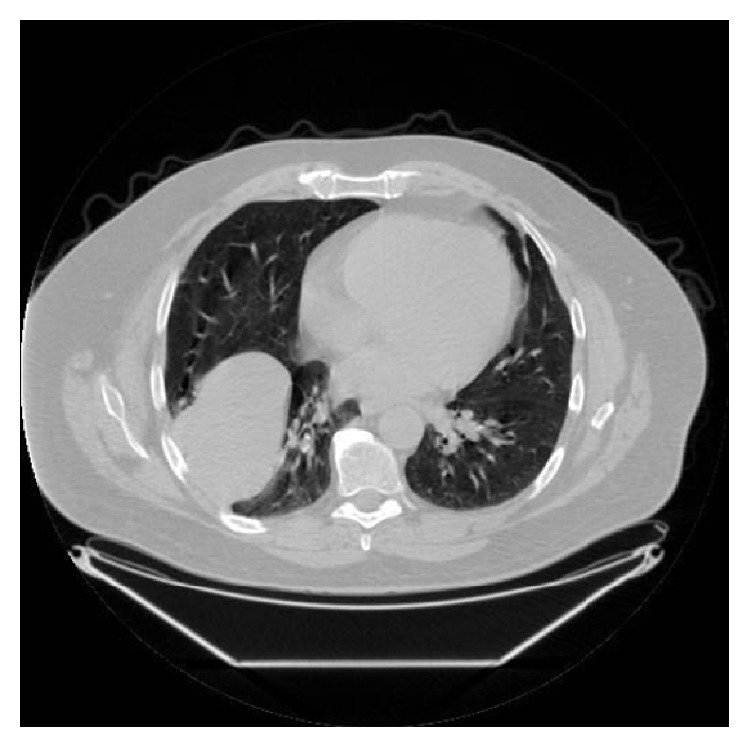
Chest computed tomography scan in lung window shows a well-circumscribed large solid mass.

**Figure 2:**
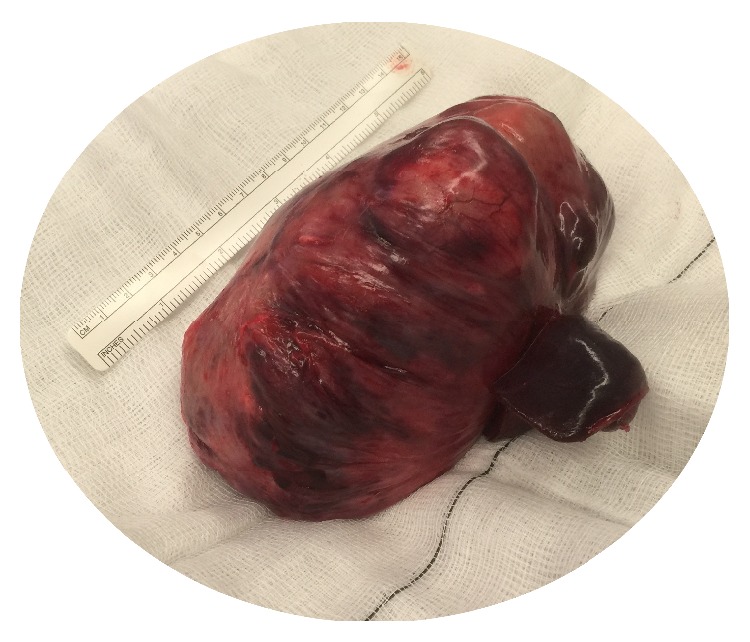
Large homogenous solid mass completely resected (macroscopic view).

**Figure 3:**
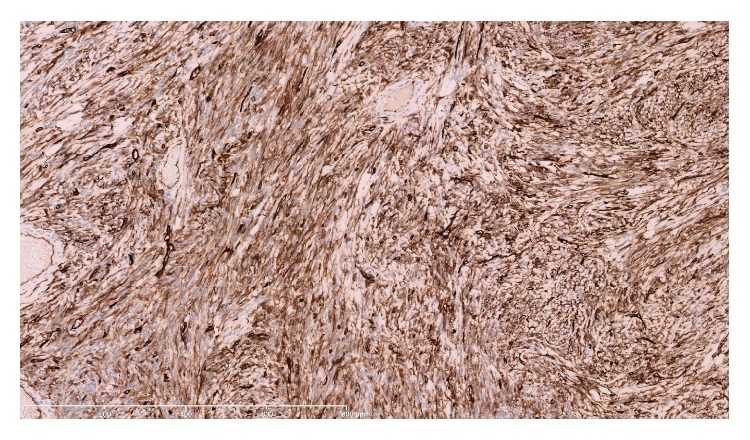
The spindle cells show strong and diffuse positivity for CD34. ×100 magnification.

**Figure 4:**
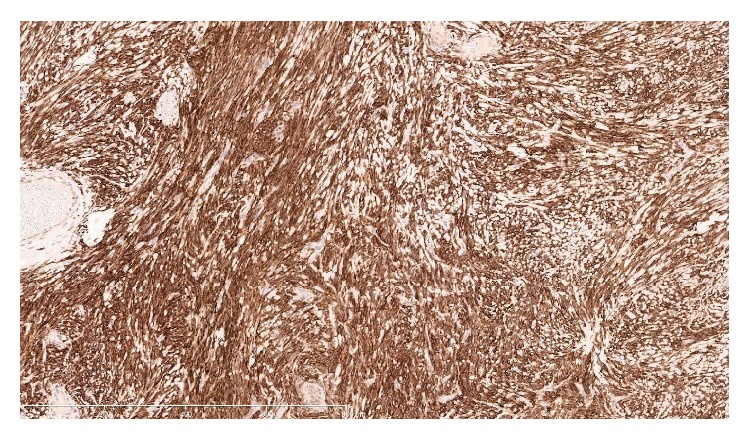
The spindle cells show strong and diffuse positivity for Bcl-2. ×100 magnification.

**Figure 5:**
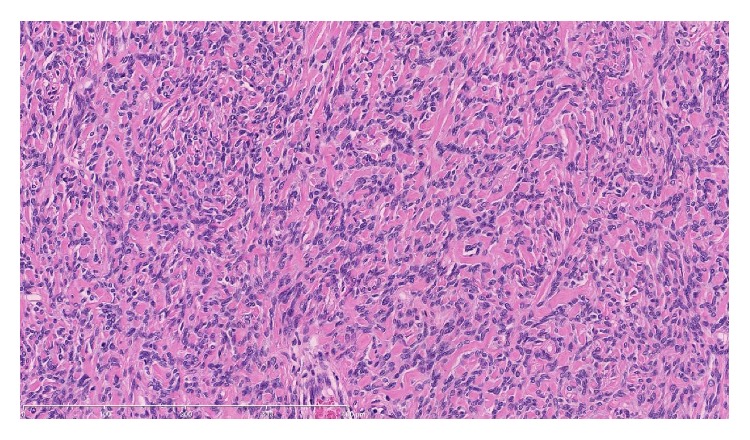
Tumour showing areas of hypocellularity and dense collagenous stroma. ×200 magnification.

**Figure 6:**
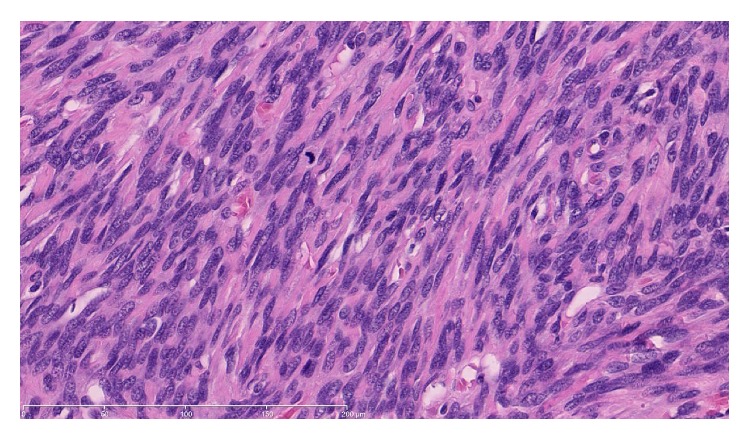
Spindle cells show marked nuclear atypia, overcrowding, and brisk mitotic activity. ×400 magnification.

A 52-year-old nonsmoking, male security worker presented with worsening dyspnoea and localised dull discomfort in the right midaxillary region between the 5th and 8th intercostal space. Routine blood tests were normal. Pulmonary function tests were normal. A computed tomography (CT) scan confirmed the presence of a solid mass measuring 14 × 7.5 × 9 cm occupying the fissure between the right middle and lower lobe (Figure 1). The mass increased in size by 75% compared with a CT scan performed a year earlier (Figure 2). The significant increase in tumour size and the onset of new symptoms led to a decision to resect the mass and establish a histological diagnosis. A right posterolateral thoracotomy was performed and a solid mass with a pedunculated attachment to the visceral pleura was found extending into the right middle and lower lobe fissure. The right lung was fully mobilised and the mass completely excised and sent for histological analysis (Figure 3). The mass was confirmed to be a solitary fibrous tumour of the pleura (SFTP) with the presence of spindle cells that tested positive for CD34, *β*-catenin, and Bcl-2 on subsequent immunohistochemistry assessment. Areas of hypercellularity, nuclear atypia, 18 mitoses per 10 high-power fields, and haemorrhagic and necrotic transformation were also identified, which confirmed that malignant transformation of a benign tumour had occurred (Figures 4, 5, and 6). The patient made an excellent postoperative recovery and was discharged home on day 4 after surgery. At 6 months' follow-up, the patient was clinically well and a repeat CT scan showed no evidence of recurrence.

## 3. Discussion

It is widely recognised that SFTPs are rare neoplasms that are predominantly benign with no gender predilection [2, 4, 5, 8, 11]. The literature suggests that between 10 and 30% of SFTPs are malignant [2, 7, 11] and display the characteristics described by England et al., that is, hypercellularity, nuclear atypia, >4 mitoses per 10 high-power fields, haemorrhage, and necrosis [2, 4, 12]. Our findings correlate with previous reports showing positive immunohistochemistry for CD34 and bcl-2. However, authors regard these as relatively nonspecific and recent studies have highlighted the importance of genotyping for NAB2-STAT6 gene fusion, which has been shown to distinguish SFTs from histological mimics [9, 13]. Fibrous tumours attached to the pleura were found to originate from the visceral pleura in two-thirds of cases and the parietal pleura in one-third of cases [5, 11]. The tumour size can range from between 1 cm and 39 cm [11] with those larger than 10 cm being malignant [11, 14, 15]. In addition to size, other macroscopic features indicating malignancy include lack of a pedicle and atypical location, for example, attachment to parietal pleura and inversion into peripheral lung [5, 11]. Our case was unusual because the tumour was attached to the visceral pleura via a pedicle—a feature consistent with benign tumours [5, 11]. Histological analysis, however, confirmed it to be a malignant SFTP based on immunohistochemistry tests and England's criteria as described earlier.

Prognosis of malignant SFTPs is often related to tumour size and remains poor with a 5-year survival rate reported to range between 45 and 68% [4, 7, 15]. Demicco et al. [14] developed a novel risk stratification model for predicting the probability of metastasis based on age, tumour size, and mitotic figures (Table 1). Based on this model, the case we presented was considered to be of* moderate* risk for metastasis. Accordingly, the follow-up for this patient included 6 monthly assessments for the first 2 years after surgical resection, which is comprised of full history, clinical examination, and CT scan. In the absence of recurrent disease at 2 years' follow-up, CT scans are performed yearly thereafter for 5 years. This case demonstrates the importance of maintaining awareness of the malignant potential of SFTPs. Furthermore this case poses an interesting question regarding the value in performing surgical excision early in the disease process in order to obtain early histological diagnosis and therefore allowing for early oncological referral and assessment.

## 4. Conclusion

We present a case of a large malignant solitary fibrous tumour of the pleura that was successfully resected. Based on a risk model for metastasis developed by Demicco et al. [14], this particular SFTP was deemed as moderate risk for metastasis. Surgery is the gold standard treatment and we propose that surgical excision be performed early in the disease process, thereby facilitating early histological diagnosis and early oncological input.

## Figures and Tables

**Table 1:** Risk stratification model [14].

Risk factor	Score
Age	
<55	0
≥55	1
Tumour size (cm)	
<5	0
5 to <10	1
10 to <15	2
≥15	3
Mitotic figures (/10 high-power fields)	
0	0
1–3	1
≥4	2
Risk	Total score
Low	0–2
Moderate	3-4
High	5-6

## References

[1] Franzen D., Diebold M., Soltermann A. (2014). Determinants of outcome of solitary fibrous tumors of the pleura: an observational cohort study. *BMC Pulmonary Medicine*.

[2] Langman G. (2011). Solitary fibrous tumour: a pathological enigma and clinical dilemma. *Journal of Thoracic Disease*.

[3] Sakurai H., Tanaka W., Kaji M., Yamazaki K., Suemasu K. (2008). Intrapulmonary localized fibrous tumor of the lung: a very unusual presentation. *The Annals of Thoracic Surgery*.

[4] Furukawa N., Hansky B., Niedermeyer J., Gummert J., Renner A. (2011). A silent gigantic solitary fibrous tumor of the pleura: case report. *Journal of Cardiothoracic Surgery*.

[5] Ludhani P., Anathakrishnan R., Muthubaskaran V., Chandrasekar P., Muralidharan S. (2015). Giant solitary fibrous tumor of the pleura. *Asian Cardiovascular & Thoracic Annals*.

[6] Chafik A., Alaoui M., Benjelloune A., Qamouss Y. (2011). A solitary fibrous tumor of the pleura revealed by hiccups. *Case Reports in Medicine*.

[7] Jeon H. W., Kwon S. S., Kim Y.-D. (2014). Malignant solitary fibrous tumor of the pleura slowly growing over 17 years: case report. *Journal of Cardiothoracic Surgery*.

[8] Yaran P., Taştepe A. I., Yazici Ü., Sak S. D. (2011). Intrapulmonary solitary fibrous tumour of the lung: a very unusual presentation. *Balkan Medical Journal*.

[9] Vogels R. J. C., Vlenterie M., Versleijen-Jonkers Y. M. H. (2014). Solitary fibrous tumor—clinicopathologic, immunohistochemical and molecular analysis of 28 cases. *Diagnostic Pathology*.

[10] Koelsche C., Schweizer L., Renner M. (2014). Nuclear relocation of STAT6 reliably predicts *NAB2-STAT6* fusion for the diagnosis of solitary fibrous tumour. *Histopathology*.

[11] Travis W. D. (2010). Sarcomatoid neoplasms of the lung and pleura. *Archives of Pathology and Laboratory Medicine*.

[12] England D. M., Hochholzer L., McCarthy M. J. (1989). Localized benign and malignant fibrous tumors of the pleura: a clinicopathologic review of 223 cases. *The American Journal of Surgical Pathology*.

[13] Doyle L. A., Vivero M., Fletcher C. D. M., Mertens F., Hornick J. L. (2014). Nuclear expression of STAT6 distinguishes solitary fibrous tumor from histologic mimics. *Modern Pathology*.

[14] Demicco E. G., Park M. S., Araujo D. M. (2012). Solitary fibrous tumor: a clinicopathological study of 110 cases and proposed risk assessment model. *Modern Pathology*.

[15] Milano M. T., Singh D. P., Zhang H. (2011). Thoracic malignant solitary fibrous tumours: a population-based study of survival. *Journal of Thoracic Disease*.

